# Novel niobium-doped titanium oxide towards electrochemical destruction of forever chemicals

**DOI:** 10.1038/s41598-021-97596-7

**Published:** 2021-09-09

**Authors:** Jesse S. Ko, Nam Q. Le, Danielle R. Schlesinger, Dajie Zhang, James K. Johnson, Zhiyong Xia

**Affiliations:** The Johns Hopkins University Applied Physics Laboratory, Laurel, MD 20723 USA

**Keywords:** Electrocatalysis, Pollution remediation, Density functional theory

## Abstract

Electrochemical advanced oxidative processes (EAOP) are a promising route to destroy recalcitrant organic contaminants such as per- and polyfluoroalkyl substances (PFAS) in drinking water. Central to EAOP are catalysis-induced reactive free radicals for breaking the carbon fluorine bonds in PFAS. Generating these reactive species electrochemically at electrodes provides an advantage over other oxidation processes that rely on chemicals or other harsh conditions. Herein, we report on the performance of niobium (Nb) doped rutile titanium oxide (TiO_2_) as a novel EAOP catalytic material, combining theoretical modeling with experimental synthesis and characterization. Calculations based on density functional theory are used to predict the overpotential for oxygen evolution at these candidate electrodes, which must be high in order to oxidize PFAS. The results indicate a non-monotonic trend in which Nb doping below 6.25 at.% is expected to reduce performance relative to TiO_2_, while higher concentrations up to 12.5 at.% lead to increased performance, approaching that of state-of-the-art Magnéli Ti_4_O_7_. TiO_2_ samples were synthesized with Nb doping concentration at 10 at.%, heat treated at temperatures from 800 to 1100 °C, and found to exhibit high oxidative stability and high generation of reactive oxygen free radical species. The capability of Nb-doped TiO_2_ to destroy two common species of PFAS in challenge water was tested, and moderate reduction by ~ 30% was observed, comparable to that of Ti_4_O_7_ using a simple three-electrode configuration. We conclude that Nb-doped TiO_2_ is a promising alternative EAOP catalytic material with increased activity towards generating reactive oxygen species and warrants further development for electrochemically destroying PFAS contaminants.

## Introduction

Per- and poly- fluoroalkyl substances (PFAS) are man-made chemicals that have a high stability in the environment due to the strength of the carbon–fluorine (C-F) bond^1^. Long-term studies reveal that PFAS can bioaccumulate differently than other contaminants owing to their high water solubility, thus entering the body through potable water and causing diverse chronic health effects^2^. Meanwhile, PFAS have dispersed globally in groundwater for over sixty years, reaching far from pollution sources^3^, and their thermal and chemical stability pose significant technical challenges for remediation. Separation-based technologies are the most common treatment method for PFAS-contaminated water, but this approach still requires destruction of the secondary PFAS waste stream, leading to other logistical, environmental, and health concerns^4^. In addition to separation methods, there is an urgent need for more effective methods of PFAS destruction.

To accelerate the identification and experimental development of novel catalytic materials for PFAS destruction, computational tools based on first-principles density functional theory (DFT) are useful means^5^. DFT-based methods have frequently been leveraged for applications relating to hydrogen and oxygen evolution reactions (HER and OER, respectively) for water splitting^6^. Wide ranges of both metals and oxides have been computationally screened and placed on “volcano plots” that exhibit a peak in catalytic activity at certain moderate binding energies of critical intermediate species^7^. Accordingly, DFT catalyst screening has generally focused on optimizing materials toward the peaks of OER and HER volcano plots^8,9^. In this work, we adapt these methods for a different purpose, i.e., to screen materials for the breakdown of recalcitrant contaminants such as PFAS. In this case, we apply DFT calculations to seek materials far from the peak in the volcano plot and hence exhibiting suppressed OER, which is a prerequisite of electrode materials for electrochemical advanced oxidative processes (EAOP)^10^.

EAOP have garnered intense interest due to the utilization of an electromotive force to induce the destruction of recalcitrant PFAS contaminants^10–14^. The key advantage of EAOP in destroying PFAS lies in its environmental compatibility, being based on electricity rather than additional chemicals that are themselves potentially toxic or require subsequent removal^10,11,15^. The underlying mechanism for contaminant destruction by EAOP has been the subject of debate, whether it is driven by direct oxidation at the electrode surface^16^ or by indirect oxidation via electrogenerated intermediates^17^. In the case of either hypothesized mechanism, an increased overpotential for OER is desired, hence suppressing it. In the first case, the rate-limiting step would be direct electron transfer between the electrode and the contaminant molecule. This mechanism has been supported in some work, for example, by comparison of experimentally measured kinetics with activation barriers calculated through DFT^16^. In the second case, the rate-limiting step is presumed to be electrochemical production of hydroxyl free radicals (^•^OH) as a byproduct of water oxidation at the anode surface, which then react unselectively with recalcitrant organic contaminants in water (Eq. 1):^18^

1 $$ {\text{H}}_{{2}} {\text{O }} \to^{\cdot} {\text{OH }} + {\text{ H}}^{ + } + {\text{ e}}^{ - } $$

This EAOP can further cascade to generate other ROS free radicals such as superoxide radical (O_2_$\bullet$^–^), hydroperoxyl radical ($\bullet$OOH), etc. (Figure S1)^10,11,18^. These free radicals possess the capability to oxidize (e.g. break) strong C–F bonds (531 kJ mol^–1^)^11^. However, due to the short lifetime of these radicals (e.g. 2–4 μs) in aqueous solutions, novel materials that can generate copious amounts of radicals are needed^11,18^. Of note, the fluoro-carbon organics’ stability demands high generation of $\bullet$OH, and only a few EAOP electrode materials have demonstrated efficacy for this difficult operation^11^.

The current state-of-the-art EAOP electrode is based on a highly oxidatively stable, conductive material, boron-doped diamond (BDD)^10,19,20^. Though diamond has a large bandgap (> 5 eV) that renders it as an electrical insulator, this material can be made semi-conductive (*p*-type semiconductor) by doping with boron atoms^17,21–23^. The stability of BDD results from carbon atoms being in *sp*^3^ hybridization; however, these electrodes are still subject to failure due to delamination from the substrate^24^. Moreover, the high cost associated with the preparation of this material has motivated the study of alternative candidates that also exhibit comparable activity and stability. The cost of diamond is the limiting factor in the usage of this EAOP electrode^24^. Therefore, alternatives such as doped-tin oxides, lead oxides, and titanium oxides have been the subject of interest for identifying cost-effective, stable, novel EAOP catalytic materials^10^.

Among those alternatives to BDD, titanium oxides exist in a number of polymorphs (e.g. rutile, anatase, and brookite), and are promising owing to their high oxidative stability, low cost, and high electrical conductivity when expressed as a defective material (e.g., typically with oxygen vacancies)^10,25–27^. Stoichiometric TiO_2_, an insulator, is an inactive electrode and is not able to participate in the generation of ROS. Conductive Magnéli titanium oxide (Ti_4_O_7_; *n*-type) has recently been implemented for PFAS destruction and has demonstrated high stability, coupled with high efficacy for destroying organic contaminants (< 10% PFAS concentration in permeate)^12,15^. Several sub-oxides of Ti_4_O_7_ phases exist, based on the generic chemical formula, Ti_n_O_2n–1_, 4 ≤ n ≤ 10; the most conductive phases are Ti_4_O_7_ and Ti_5_O_9_. Magnéli Ti_4_O_7_ is also commercially available, which ensures a seamless transition to industrial-scale applications. The key disadvantage to this material is that molecular oxygen can likely be incorporated into the lattice structure upon anodic polarization due to the oxygen deficient nature of Ti_4_O_7_, thus forming an insulating TiO_2_ layer at the surface^10,28^. Given the breadth of studies focused on understanding Ti_4_O_7_, identifying other TiO_2_-based electrodes with higher stability during anodic polarization would greatly benefit EAOP research toward applications such as PFAS destruction.

Another strategy for producing conductive TiO_2_ involves doping with group V elements such as V, Nb, and Ta, which partially converts Ti(IV) to Ti(III) and produces *n*-type semi-conductivity^29–31^. Though the possibility of producing oxygen vacancies may still exist, low dopant concentrations (< 10 at%) should yield much less vacancies as compared with Ti_4_O_7_. A prior study in 1997 by Kesselman et al. demonstrated doping TiO_2_ with niobium (Nb-TiO_2_) generated stable and conductive electrodes that can produce $\bullet$OH radicals with an anodic bias greater than 2 V *vs.* standard hydrogen electrode (SHE)^29^. Nb-TiO_2_ may therefore be an alternative EAOP catalytic material with stability, activity, and cost competitive with Magnéli Ti_4_O_7_ and BDD. However, literature on Nb-TiO_2_ for this application is scarce; thus, this material warrants further investigation on its potential use as a durable, EAOP catalytic material^10^.

Herein, we hypothesize that Nb-doped TiO_2_ electrodes may provide an effective means for PFAS destruction through the generation of ROS. We report the results of DFT calculations investigating the effect of Nb doping in TiO_2_ on its predicted electrochemical properties. These results are compared with Magnéli phase Ti_4_O_7_, which has also been shown to effectively destroy perfluorooctanoic acid (PFOA) and perfluorooctanesulfonic acid (PFOS)^12^. To confirm the DFT results, we also synthesized Nb-doped TiO_2_ and performed experimental measurements of its oxidative stability, generation of ROS, and destruction of PFOA and PFOS. The results confirm that Nb-doped TiO_2_ may be considered as a potential EAOP material for PFAS destruction.

## Results

**Figure 1:**
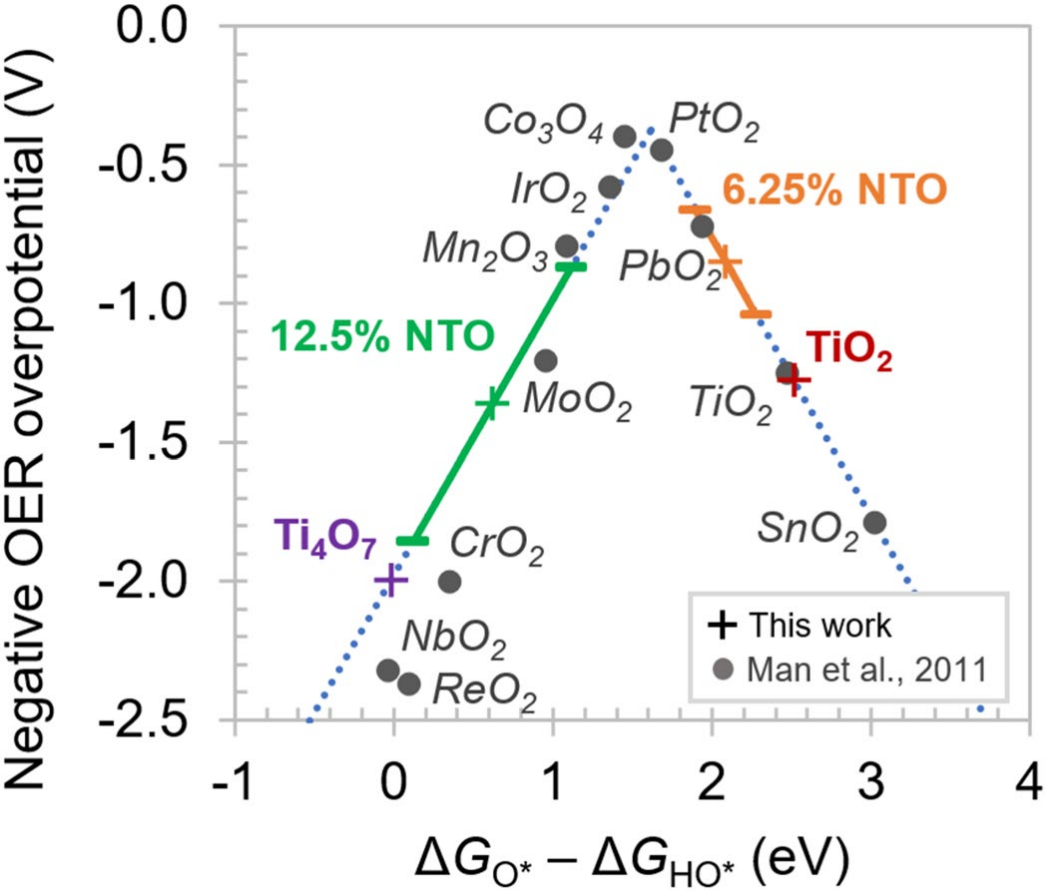
Predicted trends in oxygen evolution overpotential relative to SHE based on DFT calculations. Higher loadings of Nb-doped TiO_2_ (NTO) lead to reduction of the critical descriptor ${\Delta }G_{O*} - {\Delta }G_{HO*}$ compared to pure rutile TiO_2_. This corresponds to an initial decrease in the magnitude of the OER overpotential prior to increasing again. The suppressed OER activity of 12.5 at.% NTO is predicted to approach that of Ti_4_O_7_, which was shown previously as a candidate for PFAS destruction.

We first report the results of our DFT calculations with the goal of comparing TiO_2_-based materials for effective ROS production. As a proxy, we have predicted the activity of several materials based on established methods for the oxygen evolution reaction (OER) that must be suppressed in order to preferentially generate ROS, as shown in Figure S1. A high overpotential for OER is therefore an important criterion for catalytic materials for high ROS production^10^. This activity can be predicted in terms of a theoretical overpotential for OER based on a combined descriptor, ${\Delta }G_{O*} - {\Delta }G_{HO*}$, which is the difference between free energies of binding of the O* and HO* intermediates of the OER mechanism (asterisks denoting surface sites)^7^. The results for rutile TiO_2_, Nb-doped TiO_2_ (NTO), and Magnéli phase Ti_4_O_7_ are shown in Fig. 1, and the DFT results used to calculate the quantity ${\Delta }G_{O*} - {\Delta }G_{HO*}$ for each material are reported in Table 1. Calculated results from prior work by Man et al.^7^ are shown (gray circles) for a range of oxides, including TiO_2_ which enables verification of our calculation procedure.

**Table 1:** Total energies in eV of TiO_2_, Ti_4_O_7_, and Nb-doped TiO_2_ surfaces with O* and HO* adsorbates calculated using DFT and the resulting free energy differences used as predictor for the OER potential, $\eta$.

		6.25 at.%			12.5 at.%			
	TiO_2_	NTO (A)	(B)	(C)	NTO (A)	(B)	(C)	Ti_4_O_7_
$E_{O*} - E_{HO*}$	18.834	18.059	18.356	18.812	16.248	16.671	17.901	16.303
$E_{O*} - E_{HO*} + \frac{1}{2}E_{{H_{2} }}$	2.803	2.028	2.325	2.781	0.217	0.640	1.870	–0.272
${\Delta }G_{O*} - {\Delta }G_{HO*}$	2.513	1.738	2.035	2.491	–0.073	0.350	1.580	–0.018
$\eta$(V)	1.3	0.85 $\pm$ 0.19			1.4 $\pm$ 0.49			2.0

**Figure 2:**
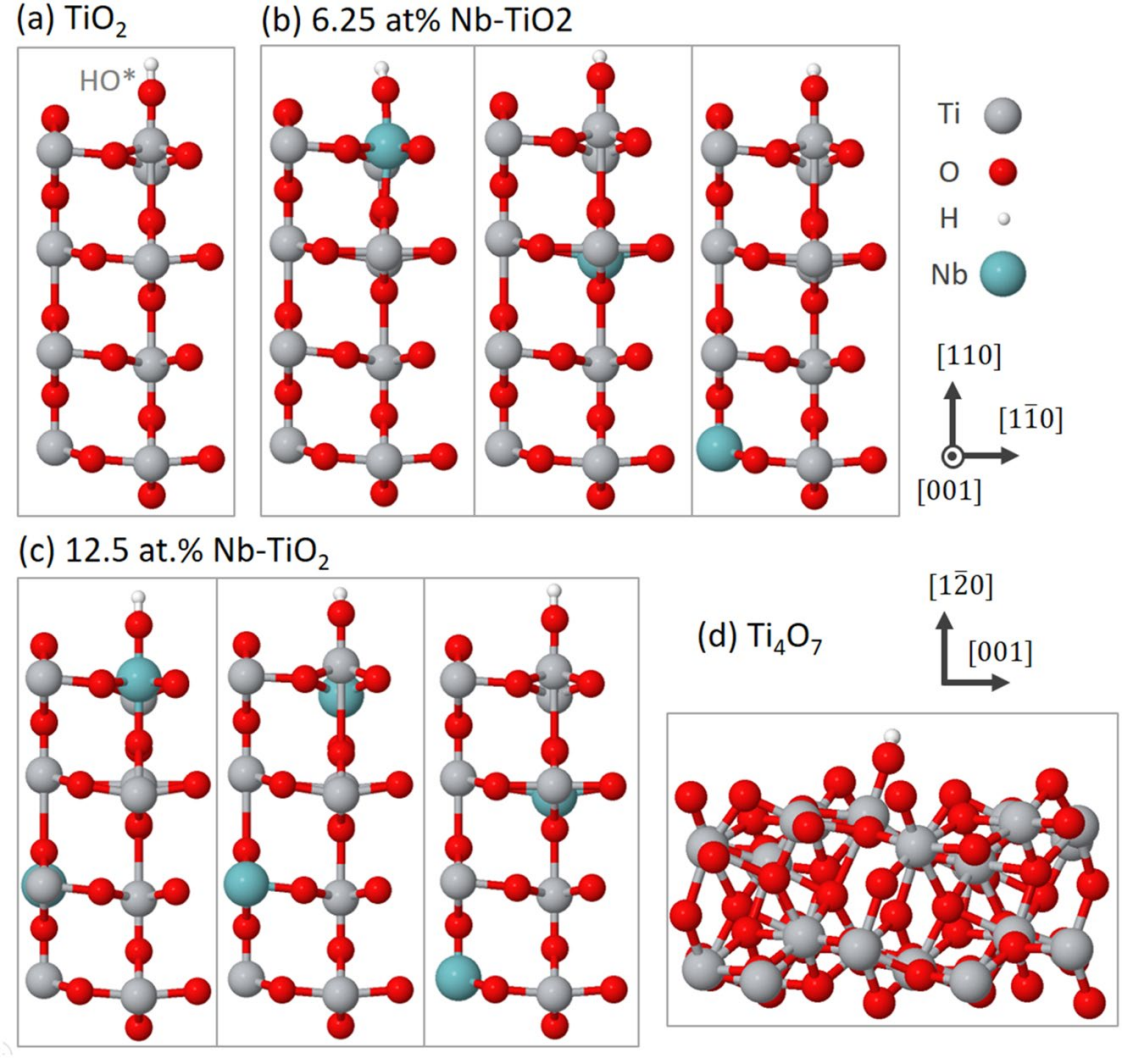
Structural models of the (**a**) TiO_2_ $\left( {110} \right)$ surface, the same surface with (**b**) 6.25 at.% and (**c**) 12.5 at.% Nb doping, and the (d) Ti_4_O_7_ $\left( {1\overline{2}0} \right)$ surface. Multiple structures were generated for each doped material system to capture the strong effect of proximity of the dopants to the surface. The models are periodic in all three directions, with 20 Å of vacuum normal to the surface. For each system, the shown geometry resulted from optimization with bound HO*. Images generated using Jmol version 14.29^40^.

In the cases of 12.5 at.% and 6.25 at.% NTO, we generated multiple model structures with the same dopant concentration in different spatial configurations relative to the surface. Recent literature has shown that modeling binding energies on surfaces of materials with random defects requires particular care in this regard^34^. The structures for all four material systems are shown in Fig. 2, including three structures shown for NTO at each of the two dopant levels. In both cases, we found that structures with Nb substituted at surface sites (A in Table 1) result in much smaller values of ${\Delta }G_{O*} - {\Delta }G_{HO*}$ than those with Nb only at subsurface sites (B and then C). The magnitude depends significantly and monotonically on the depth of the impurity atoms within the top three trilayers, with the variation diminishing to within 0.06 eV between the third and fourth trilayers. Therefore, for NTO at each loading level, the overpotential cannot be estimated from any single model structure, which would fail to capture the effects of random occupation in the experimentally synthesized materials. We therefore report the calculated values in Fig. 1 and Table 1 in terms of the mean and standard error among point calculations at the same Nb concentration.

**Figure 3:**
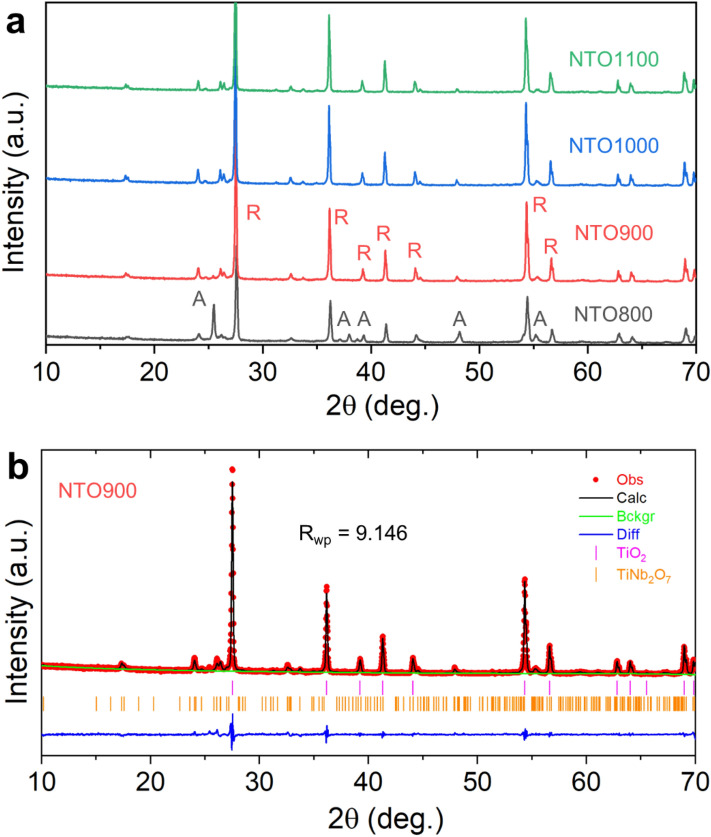
(**a**) X-ray diffraction patterns of Nb-TiO_2_ calcined at various temperatures (800, 900, 1000, and 1100 °C). A and R labels correspond go anatase and rutile phases, respectively. (**b**) Rietveld-refined X-ray diffraction pattern of NTO900 with a yielded goodness-of-fit factor of 9.146.

To validate the DFT calculations on understanding the fundamental properties of NTO, this material was first synthesized using a solution process comprising niobium(V) oxalate hydrate and titanium(IV) isopropoxide as the metal precursor sources in a stoichiometric ratio of 1:9, leading to a theoretical 10 at.% Nb dopant stoichiometry (i.e. Nb_0.1_Ti_0.9_O_*x*_). Carbon black was used as a sacrificial support to reduce agglomeration of the oxide powders. A series of post-heat treatment protocols was carried out on the as-derived powders to induce crystallization into the rutile phase (Fig. 3a). The calcination temperatures used were 800, 900, 1000, and 1100 °C, and the Nb-doped TiO_2_ samples are denoted as NTO800, NTO900, NTO1000, and NTO1100, respectively. At 800 °C, a mixture of anatase and rutile phases are present, while at 900 °C, a predominately rutile phase is observed. At elevated heat-treatment temperatures, the diffraction peaks corresponding to the rutile phase increase, thus indicating the increase in particle size. The minor impurity peaks seen in Fig. 3a for 900, 1000, and 1100 °C correspond to the titanium niobium oxide phase TiNb_2_O_7_. To quantify the phase fraction of TiNb_2_O_7_, Rietveld refinement was further performed to quantify the fractions between TiNb_2_O_7_ and rutile TiO_2_ (Fig. 3b). Based on a goodness-of-fit factor (R_wp_) of 9.146, we calculated that rutile TiO_2_ comprises 98.8% of the total diffraction pattern, while TiNb_2_O_7_ amounts only to 1.2%. Therefore, for the purposes of electrochemical characterization, we turned our attention to NTO900.

**Figure 4:**
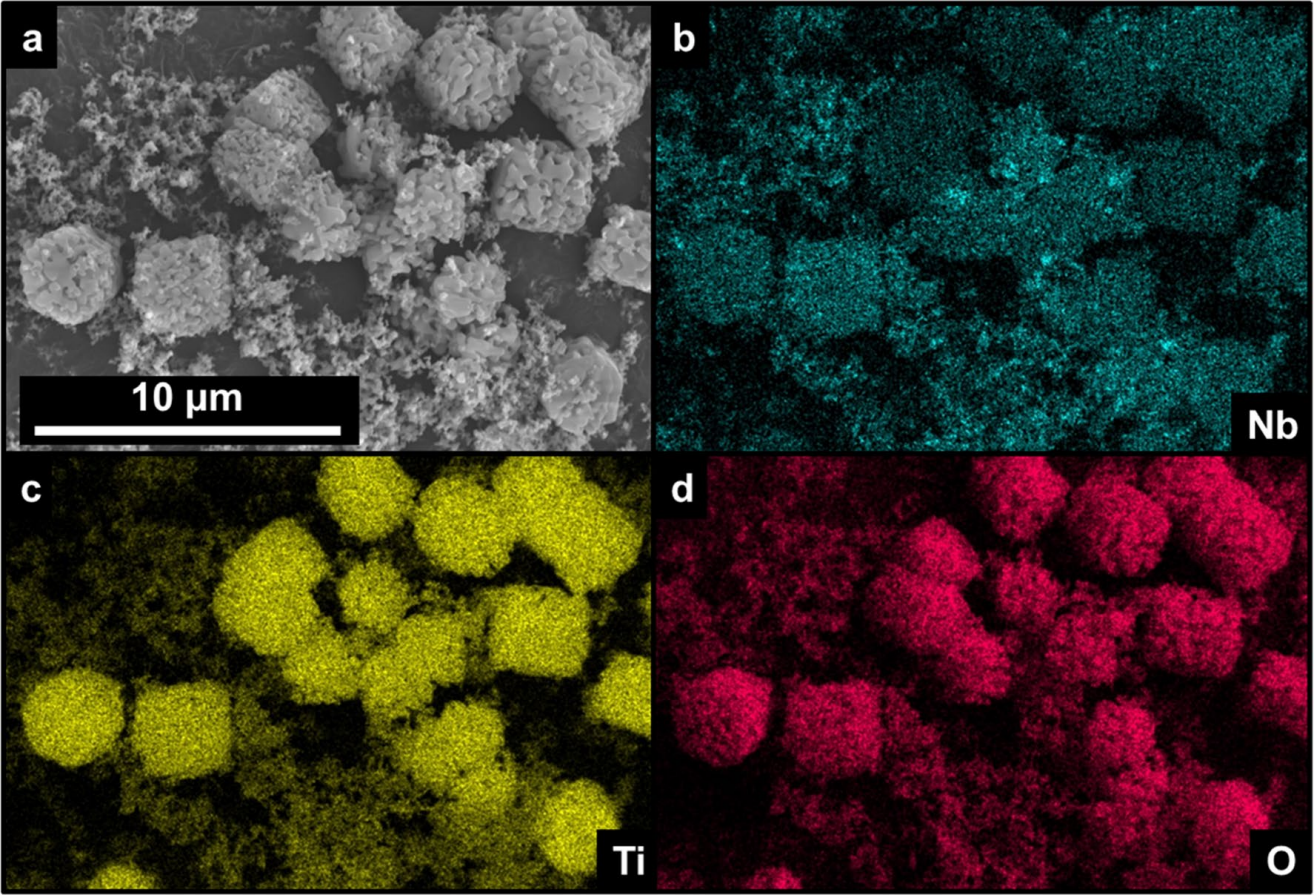
(**a**) SEM micrograph of NTO900 showing overall particle morphology coupled with EDS elemental chemical mapping taken at the Nb (**b**), Ti (**c**), and O (**d**) edges, corroborating Nb doping into TiO_2_. Images generated using AZtec (Oxford Instruments).

Because TiNb_2_O_7_ is present as an ‘impurity’ phase in the X-ray diffraction pattern, we further evaluated the distribution of Nb, while also imaging the particles using scanning electron microscopy (SEM) (Fig. 4). The SEM micrograph (Fig. 4a) of NTO900 shows a distribution of smaller (hundreds of nanometers) sized particles agglomerated that form larger primary particles on the order of 3 μm. Based on the collected scanning electron micrograph, energy-dispersive spectroscopy (EDS) was performed simultaneously to yield elemental mapping for Nb, Ti, and O shown in Figs. 4b-d. The elemental mapping confirms the primary elemental constituents are Ti and O, respectively, while also showing Nb distributed throughout the TiO_2_-rich particles. This suggests that the 900 °C heat-treatment generates a uniformly-distributed NTO material, and that the rutile pattern observed from X-ray diffraction of Fig. 3 contains Nb within the crystal structure lattice. Furthermore, we performed X-ray absorption spectroscopy (XAS), and the corresponding XANES spectra for this series of NTO materials (NTO800, NTO900, NTO1000, and NTO1100) all yield the same spectra at the Nb K-edge (Figure S2), thus validating the presence of the Nb dopants in the rutile TiO_2_ structure.

To validate the generation of ROS, we first performed chronoamperometry at a potential of 3.07 V vs. SHE (pH ~ 7.0) for 10 min (Fig. 6a,b) for Magnéli Ti_4_O_7_ and this series of NTO-based EAOP electrode materials (800, 900, 1000, and 1100 °C). The supporting electrolyte consisted of 25 μL of 20 mM 2′–7′-dichlorofluorescin diacetate (DCFH-DA), a widely-used probe for detecting ROS, in 15 mL of de-ionized water^32,33^. Further details are provided in the Methods section. After electrochemical treatment at the respective potentials, this solution was analyzed for fluorescence, which showed that both Ti_4_O_7_ and NTO900 exhibit appreciable amount of ROS. Of note, electrochemical treatment at 3.0 V for 30 min increases the fluorescence of the DCFH-DA probe dramatically for both Ti_4_O_7_ and NTO900 (Figure S3). Therefore, for the next series of experiments, a potential hold for 30 min was selected.

After validating the generation of ROS, we then performed practical electrochemical treatment of water solutions containing 20 ppb of PFOA and PFOS, the two important PFAS chemicals that are advised for monitoring by the United States Environmental Protection Agency. We performed oxidation of the solutions for both Ti_4_O_7_ and NTO900 at 2.6, 2.8, 3.1, 3.3, and 3.6 V vs. SHE for 30 min. For all tests, we used a three-electrode configuration with a flooded beaker cell.

## Discussion

The DFT results summarized in in Fig. 1 show a clear trend in terms of relative strength of O* binding, which increases to the left of the volcano plot: Ti_4_O_7_ > 12.5 at.% NTO > 6.25 at.% NTO > TiO_2_. For TiO_2_, our present calculated value of 2.51 eV is in excellent agreement with literature values, corresponding to an OER overpotential of about 1.3 V. By comparison, our calculations for Ti_4_O_7_ result in a binding energy difference of –0.02 eV, corresponding to a significantly larger OER overpotential of about 2.0 eV. To our knowledge, this is the first calculation to predict the activity of Ti_4_O_7_ for OER. The predicted value is consistent with highly substoichiometric O_vac_ producing excess carriers, which were shown in a recent work to strongly increase the relative strength of O* to HO* binding^34^.

These results suggest that NTO at sufficiently high loading levels can provide ROS generation characteristics superior to pure TiO_2_ and competitive with Ti_4_O_7_. With respect to values of the descriptor ${\Delta }G_{O*} - {\Delta }G_{HO*}$, the sequence of TiO_2_, NTO of increasing concentration, and Ti_4_O_7_ crosses over the “peak” of the volcano plot, resulting in a strongly non-monotonic trend in the OER overpotential, even accounting for the reported uncertainties in the calculation. As a result, based on a high OER overpotential as a proxy for activity for ROS generation, these calculations suggest in descending order of expected ROS generation: Ti_4_O_7_ > 12.5 at.% NTO > TiO_2_ > 6.25 at.% NTO. Notably, low Nb loading levels first exhibit reduced ROS generation due to enhanced OER relative to pure TiO_2_^10^, but further increased Nb loading is predicted to increase ROS generation.

**Figure 5:**
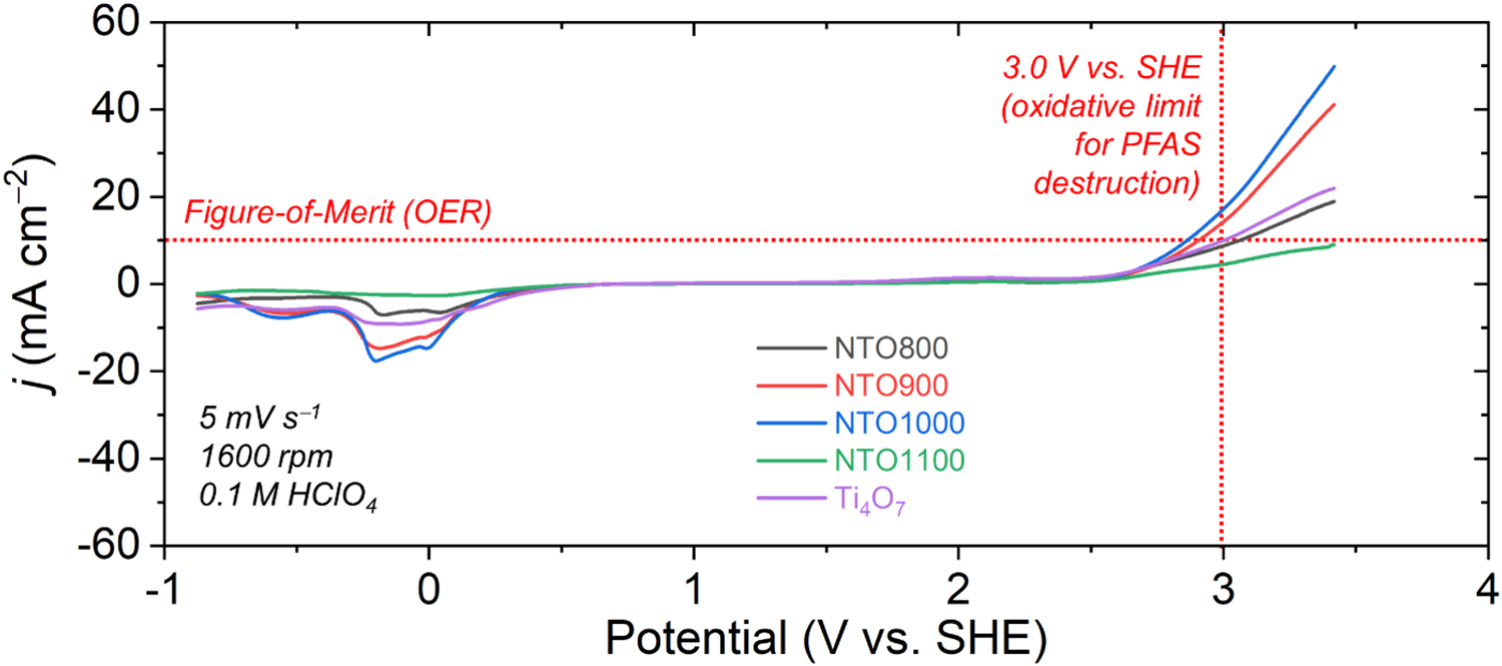
Electrochemical stability window of Ti_4_O_7_, and this series of NTO (NTO800, NTO900, NTO1000, and NTO1100) cycled with an imposed scan rate of 5 mV s^–1^, with a rotation speed of 1600 rpm, in a 0.1 M HClO_4_ supporting electrolyte. 3.0 V vs. SHE corresponds to the upper oxidative stability limit for EAOP electrodes. All electrochemical potentials were *iR* compensated for the uncompensated resistance, as determined by measuring impedance at the open circuit potential.

The results of the experimental characterization are qualitatively consistent with this picture. Initial assessment of the electrochemical stability of Ti_4_O_7_ along with this series of NTO materials was first performed to identify oxidative stability (Fig. 5). Based on a potential of 3.0 V vs. SHE, we set this as the oxidative stability limit for EAOP electrodes. Based on this threshold, all samples yield a current < 60 mA cm^–2^. Based on prior oxygen evolving catalyst studies^7,8,35^, a current density generation of 10 mA cm^–2^ yields a figure-of-merit (FOM) for OER^35^. Based on this FOM, Ti_4_O_7_, NTO800, and NTO1100 all show that OER is suppressed well below 3.0 V vs. SHE. Though NTO900 and NTO1000 showed a slightly higher current density at the FOM, these materials may still be considered promising for EAOP applications. In addition, the specific surface area of this series of NTO materials all yielded values < 9 m^2^ g^–1^, which indicates that adsorption is highly unlikely to be responsible for significant reduction of PFAS contaminants. As a control, the specific surface area of Ti_4_O_7_ was found to be 5.8 m^2^ g^–1^. Overall, the electrochemical stability window of all Ti-based materials, particularly when doped with Nb, show that OER extended into high potentials, thus indicating the likelihood for high and stable ROS generation.

**Figure 6:**
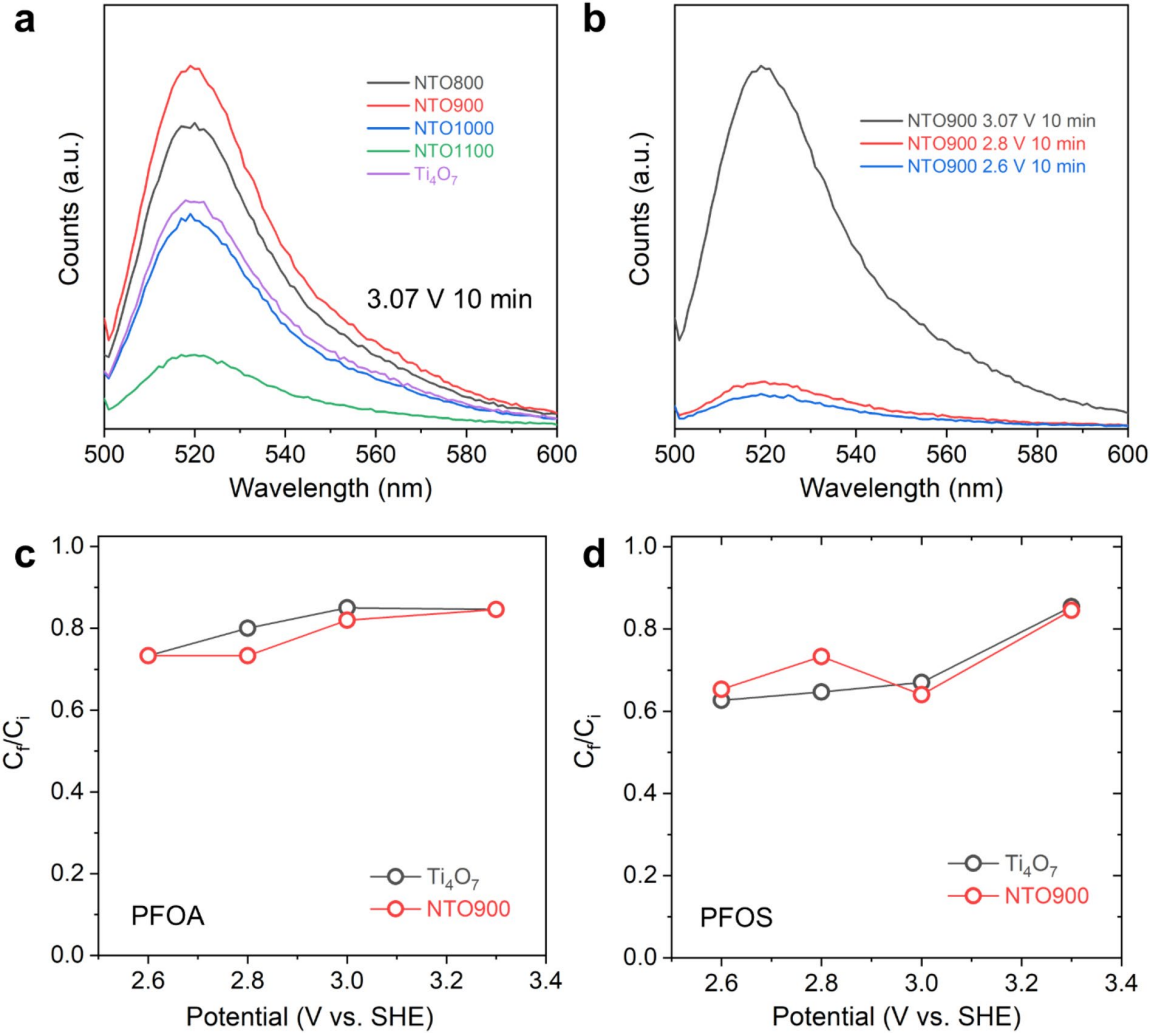
Fluorescence data using 2’–7’-dichlorofluorescin diacetate (DCFH-DA) by performing chronoamperometry at (**a**) 3.07 V vs. SHE for 10 min for NTO800, NTO900, NTO1000, NTO1100, and Ti_4_O_7_; and (**b**) 2.6, 2.8, and 3.07 V vs. SHE for 10 min of NTO900 to verify generation of reactive oxygen species. Practical electrochemical oxidation treatment of 20 ppb for 30 min of (**c**) perfluorooctanoic acid (PFOA) and (**d**) perfluorooctanesulfonic acid (PFOS) in de-ionized water for Ti_4_O_7_ and NTO900. C_f_/C_i_ is the ratio between the final and initial concentrations of the organic contaminants. Standard error for these treatments were within 5% based on replicate testing.

Qualitatively, we observe that Ti_4_O_7_ yields comparably high fluorescence, indicative of high generation of ROS compared to high calcination-derived NTO1000 and NTO1100 (Fig. 6a). With lower calcination temperatures, NTO800 and NT900 both exhibit higher fluorescence, while NTO900 is shown to generate the highest amounts of ROS. By down-selecting NTO900 as the EAOP electrode-of-interest, we then performed chronoamperometry at 2.6, 2.8, and 3.07 V vs. SHE to understand the relationship between potential and ROS generation. NTO900 achieves the highest fluorescence at 3.1 V vs. SHE and much lower fluorescence at 2.6 and 2.8 V (Fig. 6b).

For PFOA, at 2.6, 2.8, and 3.1 V, NTO900 achieved a reduction in C_f_/C_i_ to approximately 0.70, whereas Ti_4_O_7_ achieved a reduction to approximately 0.60 (Fig. 6c). At higher potentials, the C_f_/C_i_ was measured to be 0.80 for both NTO900 and Ti_4_O_7_, while NTO900 maintained that value at 3.6 V; for Ti_4_O_7_, a positive increase in C_f_ was reflected. At higher potentials, the overall increase in the C_f_/C_i_ value is attributed to the competing oxygen evolution reaction that may hinder the generation of ROS or the direct oxidation of the contaminant. For PFOS, we observe better performance for NTO900 compared to Ti_4_O_7_, where from 2.6 to 2.8 V, the C_f_/C_i_ value was ~ 0.74, while Ti_4_O_7_ gave ~ 0.8 (Fig. 6d). Similarly, at higher potentials, the reduction in PFOS concentration was not affected dramatically. Overall, the higher C_f_/C_i_ values for PFOS vs. PFOA are consistent with that observed by Le et al.^12^ Lastly, we note that a flow-through reactor is the ideal configuration for faster kinetics of PFAS electrochemical destruction, as evidenced by Carter et al.^16^ when they showed an order-of-magnitude faster decrease in organic contaminant destruction with BDD. Our present results suggest that follow-up studies with NTO in such a flow-through system are warranted.

## Conclusions

Here we report on the performance of Nb-doped TiO_2_ as a novel EAOP catalytic material, combining theoretical modeling with experimental synthesis and characterization. Our DFT calculations show that Nb doping below 6.25 at.% is expected to reduce reactive oxygen generation relative to TiO_2_, but higher concentrations up to 12.5 at.% should reverse this trend and increase performance to the point of being competitive with sub-stoichiometric Ti_4_O_7_. Nb-doped TiO_2_ samples were synthesized and found to generate ROS, therefore showing them to be a promising candidate material for EAOP. We conclude that Nb-doped TiO_2_ is a promising alternative EAOP catalytic material with increased activity relative to bare TiO_2_, lower cost than boron-doped diamond, and supporting comparable PFOA and PFOS reduction relative to sub-stoichiometric Ti_4_O_7_. These results point towards a promising direction for alternative TiO_2_-based EAOP catalyst materials via computation-guided synthesis and engineering of NTO.

## Methods

### Density functional theory (DFT) calculations

All calculations were performed using density functional theory with a plane wave basis as implemented in Quantum Espresso version 6.6^36^ using the revised Perdew–Burke–Ernzerhof functional of Hammer et al.^37^ (RPBE) because of its optimization for adsorption calculations. Core electrons were treated using the projector augmented wave method. The plane wave kinetic energy cutoff was 40 Ry and electron density cutoff was 400 Ry. Geometry optimizations were performed with energy minimized within 10^–4^ atomic units and forces within 10^–3^ atomic units.

Initial surface models were prepared using the Atomic Simulation Environment^38^ starting with the experimental bulk crystal structures and cleaving slab models with the desired surface exposed. We constructed models of the $\left( {110} \right)$ surface of rutile and $\left( {1\overline{2}0} \right)$ surface of Ti_4_O_7_, selected based on prior experimental work to be most stable surfaces of each material and therefore correspond to the greatest exposed area. The slab supercell model for rutile was constructed based on (2 × 2) tetragonal $\left( {110} \right)$ surface unit cells with a thickness of 4 TiO_2_ trilayers, resulting in a total supercell of 48 atoms with dimensions of 5.918 Å × 6.497 Å × 32.266 Å, corresponding to 20 Å of vacuum above the $\left( {110} \right)$ surface^39^. Monkhorst–Pack grid of 4 × 4 × 1 k-points was used to sample the Brillouin zone, and occupations were treated with Methfessel–Paxton smearing of 0.001 Ry to account for electron donors into conductive states in Nb-doped TiO_2_ and sub-stoichiometric Ti_4_O_7_ relative to bulk rutile. The bottom two trilayers were frozen during geometry optimization to model the relaxation of a semi-infinite surface rather than a two-sided slab. The same procedure was adapted to prepare the model of the $\left( {1\overline{2}0} \right)$ surface of Ti_4_O_7_ as well, resulting in a 14.526 Å × 7.122 Å × 24.496 Å supercell with 88 atoms. Densities of electronic states were calculated based on the optimized geometries using a denser Brillouin zone sampling of 8 × 8 × 1 k*-*points and Gaussian broadening of 0.02 Ry on the eigenvalues. Structures were visualized using Jmol version 14.29.^40^.

### Overpotential modeling

DFT calculations were used to estimate the overpotential $\eta$ for OER for four different materials: TiO_2_, 6.25 at.% Nb-doped TiO_2_ (NTO), 12.5 at.% NTO, and Ti_4_O_7_. For each material, $\eta$ in V relative to SHE was calculated as a function of the descriptor ${\Delta }G_{O*} - {\Delta }G_{HO*}$ using the “activity volcano” relationship which has been previously validated for a wide range of oxide surfaces:^39^

$$ \eta^{OER} = \left\{ {\max \left[ {\left( {{\Delta }G_{O*} - {\Delta }G_{HO*} } \right),3.2 {\text{eV}} - \left( {{\Delta }G_{O*} - {\Delta }G_{HO*} } \right)} \right]/e} \right\} - 1.23 V $$

The descriptor represents the difference in free energies of binding of the critical intermediate species O* and HO*. This is calculated based on total energies $E_{O*}^{DFT}$ and $E_{HO*}^{DFT}$ of systems consisting of the respective O* and HO* species bound to each material surface obtained using DFT:

$$ {\Delta }G_{O*} - {\Delta }G_{HO*} = E_{O*}^{DFT} - E_{HO*}^{DFT} + \frac{1}{2}E_{{H_{2} }}^{DFT} + \left( {{\Delta }ZPE - TS^{0} } \right) $$

The optimized energy of gas-phase H_2_ was also calculated using the respective DFT parameters within each system for consistent comparison. The zero-point energy and entropy corrections ${\Delta }ZPE$ and $TS^{0}$ were the same as those used in Ref.^39^.

### Materials and chemicals

Niobium(V) oxalate hydrate (Alfa Aesar 44,819, C_10_H_5_NbO_20_•*x*H_2_O), titanium(IV) isopropoxide, 97 + % (Alfa Aesar 77,115, Ti{OCH(CH_3_)_2_}_4_), oxalic acid dehydrate, 98% (Alfa Aesar A13886, C_2_H_2_O_4_$\bullet$2H_2_O), carbon black, acetylene, 50% compressed, 99.9% (Alfa Aesar 39,724), and distilled water.

### ***Synthesis of Nb***_***0.1***_***Ti***_***0.9***_***O***_***2***_

The weight fraction of Nb (in the form of Nb_2_O_5_) in niobium(V) oxalate hydrate (C_10_H_5_NbO_20_$\bullet$*x*H_2_O) was calibrated by calcining at 1100 °C in air for 8 h and the yield of Nb_2_O_5_ was 27.10%. This gives a C_10_H_5_NbO_20_$\bullet$*x*H_2_O nominal formula weight of 490.4244 g mol^–1^. In a typical procedure for the synthesis of 100 g of Nb_0.1_Ti_0.9_O*x* with a final calcination temperature of 800 °C, niobium(V) oxalate hydrate (57.5210 g, 0.1173 mol) was dissolved in hot distilled water (500 mL) to form Solution I. Solution II was prepared by adding 97% titanium isopropoxide (309.6987 g, 1.0965 mol) to water (1 L), then oxalic acid dehydrate (315 g, 2.4986 mol) was added to dissolve the precipitate. Solution III was made by mixing I and II and was added to carbon black (500 g) to form a paste. The paste was first dried at 120 °C, then heated in air to 800 °C at a ramp rate of 2 °C min^–1^. The dried paste stayed at 800 °C for several days until all the carbon black was removed. Four aliquots of 20 g each of the white powder were heat-treated in air for 8 h at temperatures of 800, 900, 1000 and 1100 °C to obtain the final products. The ramping rate to each temperature was 2 °C min^–1^.

### Materials characterization

SEM and energy dispersive X-ray spectroscopy (EDS) images were collected on a Thermo Scientific Scios DualBeam scanning electron microscope. SEM images and EDS mapping were obtained using a 5 kV accelerating voltage with an extraction current of 0.2 nA and a working distance of 7 mm. X-ray diffraction was performed using a PANalytical Empyrean diffractometer equipped with a CuKα (λ = 1.5416 Å) source to verify the phase of the synthesized Nb_0.1_Ti_0.9_O*x* powders and Magnéli Ti_4_O_7_. The X-ray diffraction patterns of Nb_0.1_Ti_0.9_O*x* powders calcined at 800, 900 1000 and 1100 °C were subsequently Rietveld-refined using the GSAS-II software package^41^. The specific surface area was measured by N_2_-porosimetry using a Quantachrome NOVA porosimeter. All samples were degassed with N_2_ under vacuum for 12 h at 150 °C before obtaining the isotherms. The Brunauer–Emmett–Teller (BET) model was used to determine the specific surface area of all samples. X-ray absorption (XAS) was utilized to verify the presence of the Nb dopant. X-ray absorption near-edge structures (XANES) analysis was conducted at the Nb K-edge on the Beamline for Materials Measurement (6-BM) at the National Synchrotron Light Source II at Brookhaven National Laboratory (Upton, NY). The powders were spread as a thin-film onto Kapton tape and covered with x-ray clean polyfilm. Nb XANES were collected for each sample from −100 to −20 eV below the Nb K-edge (5.0 eV step size), from −20 to 50 eV above the Nb K-edge (0.2 eV step size), and 50 eV to 15 Å^–1^ above the Nb K-edge (0.05 Å^–1^ step size) all with a 0.5 s point^–1^ acquisition time. Spectra were collected in both transmission and fluorescence modes, and a standard ionization chamber and a four element silicon drift detector were used for each of these measurement types, respectively. Nb XANES standard spectra of Nb foil and NbO_2_ were collected and used for calibration. Data processing was performed using the Athena software package^42^. Spectra were normalized by fitting a first-order polynomial to the pre-edge region and by fitting a second order polynomial to normalize the post-edge region to 1.0.

### Electrochemical characterization

Electrochemical stability tests of this series of materials was carried out using a Gamry Reference 3000 potentiostat using a three-electrode configuration with 0.1 M HClO_4_ as the supporting electrolyte (pH ~ 1.0). Platinum wire was used as the counter electrode, while all potentials were referenced to a frit-isolated Ag/AgCl electrode (+ 199 mV vs. NHE; saturated KCl). Glassy carbon (GC) electrodes (5 mm dia., geometric area = 0.196 cm^2^, PEEK casing; Gamry Instruments) were coated with a thin, uniform film of this series of Nb_0.1_Ti_0.9_Ox materials (800, 900, 1000, and 1100 °C) and Ti_4_O_7_. The film was deposited from a solution comprising 10 mg of electrocatalyst powder dispersed in a 5 wt% styrene-butadiene rubber (SBR) solution in water. An SBR polymer binder was selected, owing to its stability with the catalyst materials, as well as it being a F-free binder, which would eliminate any concerns of F leaching into the PFAS-destructed solutions. Before coating, the GC electrodes were polished with 0.5 μm alumina slurry, and thoroughly sonicated sequentially in de-ionized H_2_O and methanol, then dried under ambient conditions. Current–potential measurements for the oxidative stability of the Ti-based electrocatalysts were obtained using linear sweep voltammetry (LSV) with an imposed scan rate of 5 mV s^–1^ from 0 to 2.8 V vs. Ag/AgCl and 0 to −1.5 V vs. Ag/AgCl for assessing oxidative stability and reductive stability, respectively. All electrochemical potentials were *iR* compensated to account for the uncompensated resistance, as determined by measuring impedance at the open circuit potential.

To verify the generation of ROS, such as hydroxyl radicals, electrodes were prepared by drop-casting the same solution used to prepare GC electrodes, but at a higher mass loading, and on a 1 cm × 2 cm titanium foil substrate, with 1 cm^2^ being the electroactive geometric area. The optimized mass loading of the electrodes was obtained by drop-casting 60 μL aliquots of the solution comprising 10 mg of electrocatalyst dispersed in 5 wt% SBR, which led to a mass loading of ~ 0.2 mg cm^–2^. Chronoamperometry was performed where potentials of 2.6, 2.8, and 3.07 V vs. SHE (1.977, 2.117, and 2.447 V vs. Ag/AgCl; pH ~ 7.0) were imposed for 10, 30, and 60 min. The supporting electrolyte consisted of 25 μL of 20 mM 2′–7′-dichlorofluorescin diacetate (DCFH-DA), a widely-used probe for detecting ROS^32,33^, in 15 mL of de-ionized water. After electrochemical treatment at the respective potentials, this solution was analyzed for fluorescence using a Horiba Scientific Fluoromax + spectrometer. Each sample was placed into a 10 mL crystal cuvette and analyzed at an excitation wavelength (λ_ex_) of 495 nm (slit 5 nm) and emission wavelengths (λ_em_) from 500–600 nm (slit 2 nm).

### PFAS destruction analysis

After down-selecting for the intended electrochemical operations (constant potential 2.6, 2.8, 3.1, 3.3, and 3.6 V vs. SHE for 30 min), challenge water comprising 20 ppb of PFOA and PFOS, separately, were used as the supporting electrolyte. Using a three-electrode configuration, 15 mL of PFOA or PFOS solution was poured into a 50 mL three-neck round bottom flask. After electrochemical treatment, this solution was then poured into a 250 mL plastic bottle, and was further diluted to 200 mL with de-ionized water for analysis of initial PFAS concentration prior to treatment (C_i_) and final concentration after treatment (C_f_). Standard error of the C_f_/C_i_ values for these treatments were within 5% based on replicate testing.

## Supplementary Information

Supplementary Information.
