# Author Correction: Novel niobium-doped titanium oxide towards electrochemical destruction of forever chemicals

**DOI:** 10.1038/s41598-021-98715-0

**Published:** 2021-09-20

**Authors:** Jesse S. Ko, Nam Q. Le, Danielle R. Schlesinger, Dajie Zhang, James K. Johnson, Zhiyong Xia

**Affiliations:** The Johns Hopkins University Applied Physics Laboratory, Laurel, MD 20723 USA

Correction to: *Scientific Reports* https://doi.org/10.1038/s41598-021-97596-7, published online 09 September 2021

Dajie Zhang was omitted from the author list in the original version of this Article.

The Author Contributions section now reads:

“J.S.K. led the electrochemical characterization of the catalysts. N.Q.L. performed the theoretical calculations. D.R.S. assisted with PFAS analysis. J.K.J. contributed to data interpretation. D.Z. contributed to the synthesis of catalysts. Z.X. was responsible for conceiving and planning of the project. All authors contributed to drafting of the manuscript.”

The Acknowledgements section now reads:

“The authors acknowledge the support from the Independent Research and Development (IRAD) Fund from the Research and Exploratory Development Mission Area of the Johns Hopkins University Applied Physics Laboratory. The authors also thank Dr. Tim Montalbano for the assistance with SEM. The XANES work used the Beamline for Materials Measurement (6-BM) of the National Synchrotron Light Source II, a U.S. Department of Energy (DOE) Office of Science User Facility operated for the DOE Office of Science by Brookhaven National Laboratory under Contract No. DE-SC0012704.”

The original Article has been corrected.

