# Supplementary material for: Novel niobium-doped titanium oxide towards electrochemical destruction of forever chemicals
Source: Sci Rep. 2021 Sep 9;11:18020. doi: 10.1038/s41598-021-97596-7 (PMC8429446; doi:10.1038/s41598-021-97596-7)
Supplement: Supplementary file 1 — Supplementary Information. [file 41598_2021_97596_MOESM1_ESM.docx]

*Supporting Information*

Novel Niobium-doped Titanium Oxide Towards Electrochemical Destruction of Forever Chemicals

Jesse S. Ko,* Nam Q. Le,* Danielle R. Schlesinger, Dajie Zhang, James K. Johnson, Zhiyong Xia**

The Johns Hopkins University, Applied Physics Laboratory, Laurel, MD 20723 USA

*Equal contribution as first authors

**Corresponding author: [zhiyong.xia@jhuapl.edu](mailto:zhiyong.xia@jhuapl.edu)

**
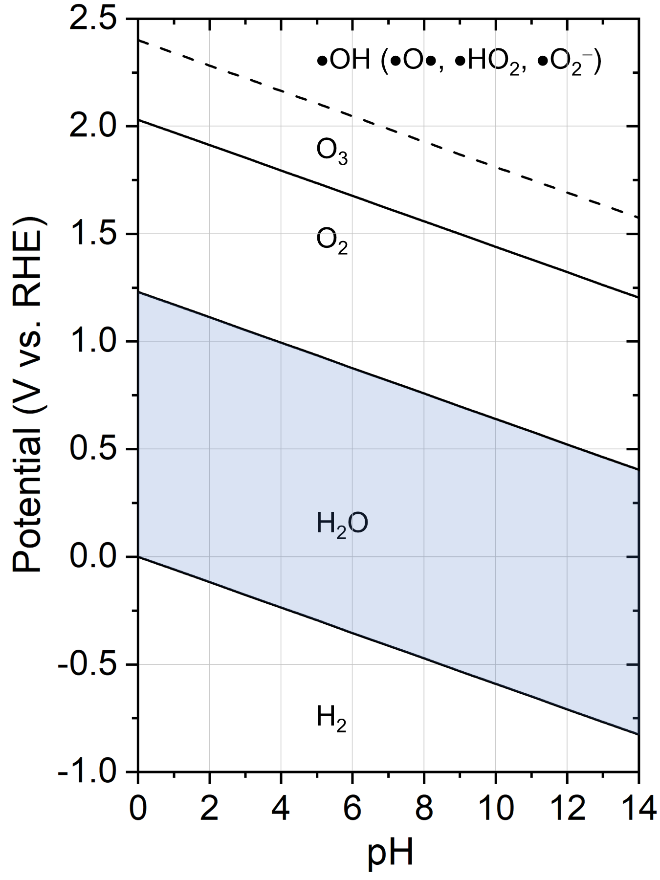
**

**Figure S1.** Potential-dependent Pourbaix diagram of water. Each line represents the overpotentials required for the generation of intermediate O_2_, H_2_, or reactive oxygen species (ROS). The dashed line at high overpotentials indicates the generation of ROS, which exhibit short lifetimes.





**Figure S2.** XANES spectra collected at the Nb K-edge for this series of NTO (NTO800, NTO900, NTO1000, and NTO1100. All normalized spectra show comparable features; thus, verifying the presence of Nb in this series of NTO samples.


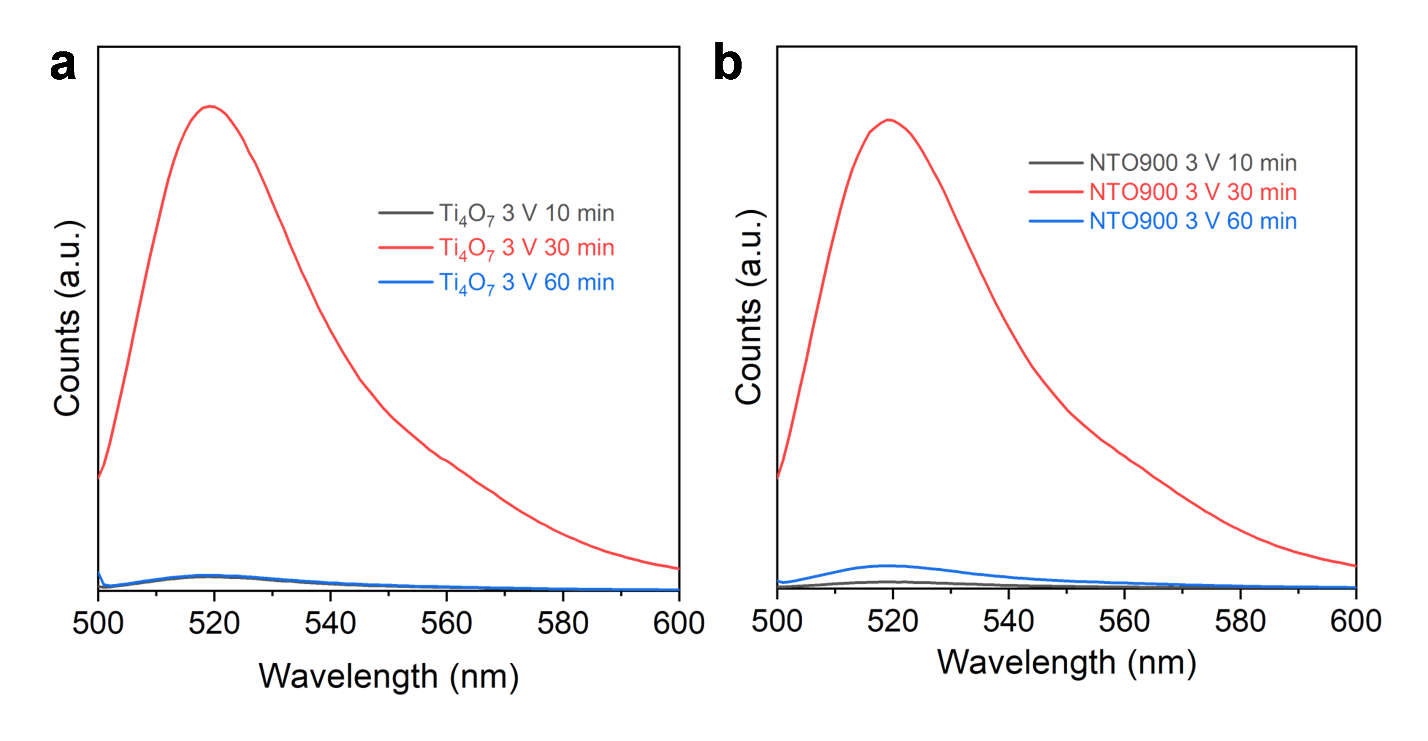


**Figure S3.** Fluorescence data using 2’-7’-dichlorofluorescin diacetate (DCFH-DA) by performing chronoamperometry at 3.07 V vs. SHE for 10 30, and 60 min for **(a)** Ti_4_O_7_ and **(b)** NTO900 to verify optimum time for generation of reactive oxygen species.
